# Longitudinal Changes in Neuromelanin MRI Signal in Parkinson's Disease: A Progression Marker

**DOI:** 10.1002/mds.28531

**Published:** 2021-03-10

**Authors:** Rahul Gaurav, Lydia Yahia‐Cherif, Nadya Pyatigorskaya, Graziella Mangone, Emma Biondetti, Romain Valabrègue, Claire Ewenczyk, R. Matthew Hutchison, Jesse M. Cedarbaum, Jean‐Christophe Corvol, Marie Vidailhet, Stéphane Lehéricy

**Affiliations:** ^1^ Paris Brain Institute– ICM Center for NeuroImaging Research – CENIR Paris France; ^2^ ICM, Sorbonne University, UPMC Univ Paris 06, Inserm U1127, CNRS UMR Paris France; ^3^ ICM Team “Movement Investigations and Therapeutics” (MOV'IT) Paris France; ^4^ Department of Neuroradiology Pitié‐Salpêtrière Hospital, AP‐HP Paris France; ^5^ INSERM, Clinical Investigation Center for Neurosciences, Pitié‐Salpêtrière Hospital Paris France; ^6^ Department of Neurology Pitié‐Salpêtrière Hospital, AP‐HP Paris France; ^7^ Biogen Inc. Cambridge Massachusetts USA

**Keywords:** neuroimaging, biomarker, neurodegenerative disorders, parkinsonism, substantia nigra

## Abstract

**Background:**

Development of reliable and accurate imaging biomarkers of dopaminergic cell neurodegeneration is necessary to facilitate therapeutic drug trials in Parkinson's disease (PD). Neuromelanin‐sensitive MRI techniques have been effective in detecting neurodegeneration in the substantia nigra pars compacta (SNpc). The objective of the current study was to investigate longitudinal neuromelanin signal changes in the SNpc in PD patients.

**Methods:**

In this prospective, longitudinal, observational case–control study, we included 140 PD patients and 64 healthy volunteers divided into 2 cohorts. Cohort I included 99 early PD patients (disease duration, 1.5 ± 1.0 years) and 41 healthy volunteers analyzed at baseline (V1), where 79 PD patients and 32 healthy volunteers were rescanned after 2.0 ± 0.2 years of follow‐up (V2). Cohort II included 41 progressing PD patients (disease duration, 9.3 ± 3.7 years) and 23 healthy volunteers at V1, where 30 PD patients were rescanned after 2.4 ± 0.5 years of follow‐up. Subjects were scanned at 3 T MRI using 3‐dimensional T1‐weighted and neuromelanin‐sensitive imaging. Regions of interest were delineated manually to calculate SN volumes, volumes corrected by total intracranial volume, signal‐to‐noise ratio, and contrast‐to‐noise ratio.

**Results:**

Results showed (1) significant reduction in volume and volume corrected by total intracranial volume between visits, greater in progressing PD than nonsignificant changes in healthy volunteers; (2) no significant effects of visit for signal intensity (signal‐to‐noise ratio); (3) significant interaction in volume between group and visit; (4) greater volume corrected by total intracranial volume at baseline in female patients and greater decrease in volume and increase in the contrast‐to‐noise ratio in progressing female PD patients compared with male patients; and (5) correlations between neuromelanin SN changes and disease severity and duration.

**Conclusions:**

We observed a progressive and measurable decrease in neuromelanin‐based SN signal and volume in PD, which might allow a direct noninvasive assessment of progression of SN loss and could represent a target biomarker for disease‐modifying treatments. © 2021 The Authors. *Movement Disorders* published by Wiley Periodicals LLC on behalf of International Parkinson and Movement Disorder Society

Parkinson's disease (PD) is characterized by progressive loss of dopaminergic neurons in substantia nigra pars compacta (SNpc).^1^ Motor symptoms in PD develop when the decrease in dopaminergic (DA) neurons reach a threshold of 30% to 60%.^2^, ^3^, ^4^, ^5^, ^6^ Treatments for PD are aiming at compensating for the loss of dopamine. Although to date, no therapeutic approaches have proven to slow disease progression, attempts have been made to identify valid imaging outcome measure for future therapeutic trial. The most studied imaging biomarker is dopamine transporter (DAT) with single photon emission computed tomography ([^123^I] FP‐CIT SPECT), DAT imaging not only reflects dopamine deficiency but also may be affected by up‐ or downregulation processes in PD.^7^, ^8^

SNc dopaminergic neurons contain a neuromelanin (NM) pigment that has paramagnetic properties.^9^ Using NM‐sensitive imaging, the SNpc shows high signal intensity related to the NM‐iron compound. Studies have reported reduced size and signal intensity in PD using NM‐sensitive imaging with high diagnostic accuracy^10^, ^11^, ^12^ predominating in the posterolateral SN.^13^ NM‐sensitive MRI has been validated histologically as a marker of NM.^10^, ^14^, ^15^, ^16^, ^17^

Longitudinal variations in the NM MRI signal in PD are poorly known, as they were only investigated in a small number of patients showing a longitudinal decrease in SN area.^18^

We investigated NM signal changes in PD patients associated with disease progression and explored its potential value as a biomarker of disease modification in clinical neuroprotective trials.

## 1 Materials and Methods

### 1.1 Subjects

We prospectively studied early PD patients (cohort I) recruited from May 2015 to February 2020 and progressing PD patients (cohort II) recruited between April 2010 and September 2012. The inclusion criteria were clinical diagnosis of idiopathic PD made by a movement disorder specialist according to the Queen Square Brain Bank criteria,^19^ aged between 18 and 75 years, and no/minimal cognitive disturbances (Mini–Mental State Examination > 24). For cohort I, disease duration was <4 years. Healthy volunteers (HVs) were included for both cohorts. The local ethics committee approved both studies, and all subjects provided written informed consent (CPP Paris VI, RCB: 2009‐A00922‐55 for Nucleipark and RCB 2014‐A00725‐42 for Iceberg).

### 1.2 Clinical Examination

The MDS‐UPDRS scale was used for cohort I and UPDRS for cohort II. To harmonize the 2 groups of patients, part III UPDRS scores were converted to MDS‐UPDRS using standard guidelines.^20^

For calculating the disease duration in years, we used the date of diagnosis as the starting point.

### 1.3 MRI Data Acquisition

For both cohorts, the MRI protocol included whole‐brain 3‐dimensional (3‐D) T1‐weighted imaging and axial turbo spin echo 2‐dimensional T1‐weighted NM‐sensitive imaging with a field of view restricted to midbrain (NM‐sensitive) at 3 T (Siemens, Erlangen, Germany).

For cohort I, subjects were scanned using a PRISMA scanner and a 64‐channel receive head‐only coil. Three‐dimensional T1‐weighted images were acquired using a sagittal Magnetization Prepared 2 Rapid Gradient Echo (MPRAGE) with a 1‐mm isovoxel size,^21^ and NM‐sensitive images were acquired with the following parameters: TR/TE/flip angle, 890 milliseconds/13 milliseconds/180°, 3 averages; voxel size, 0.4 × 0.4 × 3 mm^3^; acquisition time (TA), 6:55 minutes.

In cohort II, subjects were scanned using a TRIO 32‐channel TIM system using a 12‐channel receive head‐only coil. Three‐dimensional T1‐weighted scans were acquired using a MPRAGE with a 1‐mm isovoxel size, and NM‐sensitive image parameters were TR/TE/flip angle, 900 milliseconds/15 milliseconds/180°, 3 averages; voxel size, 0.4 × 0.4 × 3 mm^3^; TA, 6:59 minutes.

### 1.4 Image Analysis

All analyses were performed using software programs written with in‐house algorithm in MATLAB (vR2017b; MathWorks Inc, Natick, MA) and with Statistical Parametric Mapping (SPM12, UK), FreeSurfer (v5.3.0; MGH), and FSL (v5.0; FMRIB, UK).

#### 1.4.1 Region of Interest Selection

**FIG. 1:**
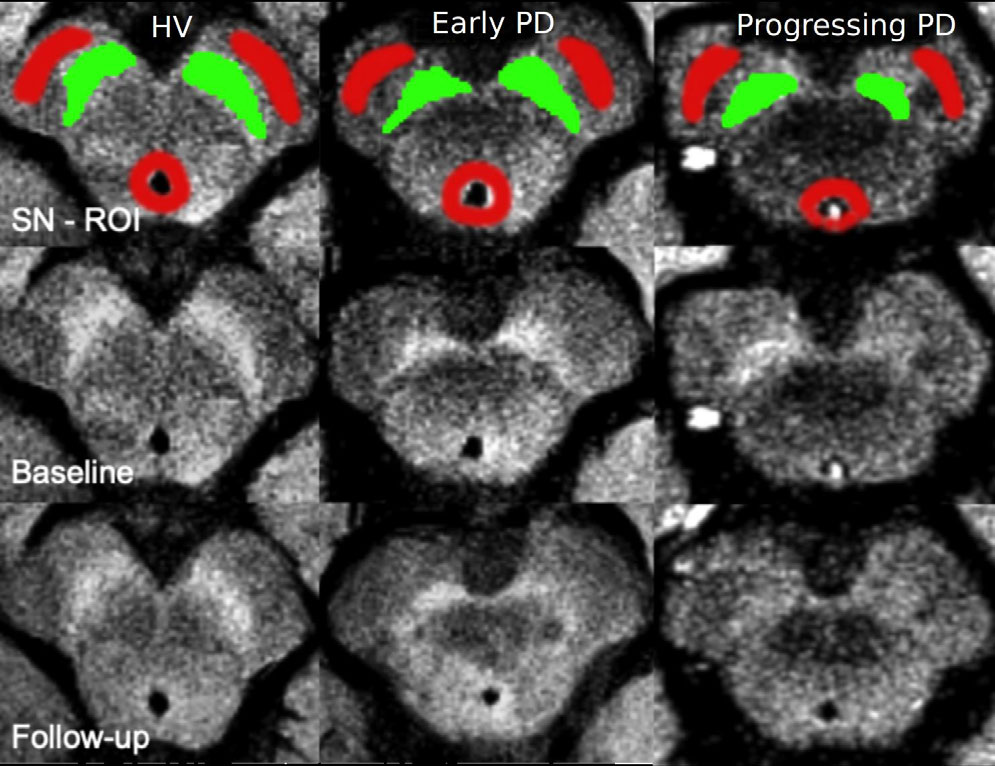
Manual tracing of the SN in neuromelanin‐sensitive images: Neuromelanin images of a representative healthy volunteer (HV, leftmost column), an early PD patient (Early PD, middle column), and a progressing PD patient (Progressing PD, rightmost column). The upper row shows the baseline images (V1) with the manual tracing of the left and right SN region of interest (ROI; SN ROI in green and background ROI in red) superimposed on the neuromelanin‐sensitive image. The middle row shows the same images without the regions of interest (baseline). The lower row shows the follow‐up images of the same subjects at V2 (follow‐up). [Color figure can be viewed at wileyonlinelibrary.com]

Using FreeSurfer viewer, SN contours were manually delineated on NM‐sensitive images by 2 independent examiners as the border of hyperintense area dorsal to the cerebral peduncle and ventral to the red nucleus manually (as in reference 22 (Fig. 1). Contours were continuous, as they did not include noncontiguous voxels. Both examiners were blind to the group (PD, HV) and visit (V1, V2) of the subject. Segmentations of V1 and V2 examinations were done side by side at the same time. Examiner 1 segmented all scans (n = 345), of which 40 scans were segmented twice on separate sessions to assess intraexaminer variability of measurements. To assess interexaminer variability of measurements, examiner 2 segmented 248 scans. Statistical analyses were done on the segmentations drawn by examiner 1, who segmented all subjects. A background region was also manually traced that included the tegmentum and superior cerebral peduncles (Fig. 1).

#### 1.4.2 Quantitative Analysis

SN volumes (Vol) were calculated using an in‐house MATLAB algorithm as the number of voxels in NM‐based regions of interest (ROIs) of the 3 lowest contiguous image slices in which the SN was visible multiplied by voxel size. Total intracranial volume (TIV) was estimated to correct for variations in individual head sizes using SPM12. White matter, gray matter, and cerebrospinal fluid volumes were summed up to provide an estimate of TIV (Table S2). We calculated corrected volume (C_vol_) by dividing SN volumes by TIV to normalize for respective head sizes of the subjects. For each slice, signal‐to‐noise ratio (SNR) and contrast‐to‐noise ratio (CNR) were calculated by normalizing the mean signal in SN relative to the background signal using the following formulas.^23^

$$\mathrm{SNR}=\text{mean}\_\text{over}\_\text{slices}\left(\left[{\mathrm{Sig}}_{\mathrm{SN}}/{\mathrm{Sig}}_{\mathrm{BND}}\right]\times 100\right])$$

$$\mathrm{CNR}=\text{mean}\_\text{over}\_\text{slices}\left(\left[{\mathrm{Sig}}_{\mathrm{SN}}\;-{\mathrm{Sig}}_{\mathrm{BND}}\right]/{\mathrm{STD}}_{\mathrm{BND}}\right)$$

where Sig_SN_ is the signal intensity in SN ROI, Sig_BND_ the signal intensity in background ROI, and STD_BND_ the standard deviation in background ROI.

A total of 34 scans over V1 and V2 (8.9%) were not analyzed, 12 because of poor scan quality or incomplete examination, 14 because of mostly head motion, 8 because of the presence of exclusion criteria (abnormal neurological or neuropsychological examinations: 4 HVs, conversion to dementia with Lewy body and corticobasal degeneration: 2, consent withdrawal due to personal reasons unrelated to the study: 1, adverse event: 1.

### 1.5 Statistical Analyses

Statistical analyses were performed using R (R Core Team 2019, v3.6.1) and MATLAB vR2017b. Clinical and demographic variable comparisons were done with parametric Student *t* tests, whereas the chi‐square test was used for sex proportions.

Imaging data were normally distributed according to the Shapiro–Wilk normality test. Hence, parametric tests were used.

#### 1.5.1 Cross‐Sectional Analysis

A 2‐way multivariate general linear model (GLM)–analysis of variance (ANOVA) was conducted with group (PD, HV) and sex as between‐group factors and age as a covariate to test for baseline between‐group difference in Vol, C_vol_, SNR, and CNR as well as interactions. Then we also conducted a separate sex‐based analysis with group (PD, HV) as the only between‐group factor while treating both sex and age as covariates. A diagnostic value was calculated using receiver operating characteristic (ROC) analysis.

#### 1.5.2 Longitudinal Analysis

We used the lm function via the mixlm R package to fit a mixed (between‐ and within‐factor) design multivariate GLM estimated using the restricted maximum likelihood ratio test for both cohorts. In cohort I, a mixed 2 × 2 × 2 multivariate GLM‐ANOVA with visit (V1, V2) as a within‐subject factor, group (PD, HV) and sex as between‐group factors, and age as a covariate was performed for Vol, C_vol_, SNR, and CNR. Then we conducted a separate sex‐based analysis with visit (V1, V2) as a within‐subject factor, group (PD, HV) as a between‐group factor while treating both sex and age as covariates.

In cohort II, a mixed 2 × 2 GLM‐ANOVA was conducted with visit as a within‐subject factor and sex as a between‐group factor while adjusting for age as a covariate, because only PD patients underwent V2 in cohort II. Here as well, we conducted a separate sex‐based analysis with visit (V1, V2) as a within‐subject factor while treating both sex and age as covariates.

Average annual rates of decline for SN measurements were computed by dividing percentage of changes between the visits by the delay between the visits.

Inter‐ and intraobserver variability was estimated using DICE and intraclass coefficients (ICCs).

Scanner effect was studied in the HV group at baseline for Vol, C_vol_, SNR, CNR, and TIV using a 1‐way ANOVA with scanner as a between‐group effect.

Pearson's correlation coefficients were calculated between SN measurements and clinical scores along with age at baseline. To adjust for multiple comparisons, an approximate multivariate permutation test was conducted. Sampling distribution was built to calculate the corrected *P* value as the proportion of values that were larger than the observed correlation coefficient value.^24^

To assess the efficiency of NM‐sensitive MRI to evaluate longitudinal changes in SN NM content, we calculated the required sample size at 80% and 90% power assuming 30%, 50%, and 70% of predicted changes.

All results were represented as mean ± standard deviation.

## 2 Results

### 2.1 Clinical Characteristics (Table 1)

**TABLE 1:** Demographic and clinical characteristics of cohorts I and II

	Healthy volunteers			Parkinson's disease patients		
	All HV	HV with follow‐up		All PD	PD with follow‐up	
	V1	V1	V2	V1	V1	V2
Cohort I						
Number of subjects	41	32	32	99	79	79
Mean age (years)	60.3 ± 9.1	61.6 ± 8.9	63.6 ± 8.9	62.0 ± 9.4	61.8 ± 9.0	63.8 ± 9.0
Male/demale	17/24^a^	11/21	11/21	66/33	49/30	49/30
MDS‐UPDRS‐III OFF score	5.7 ± 5.8^b^	6.1 ± 6.2	6.8 ± 5.8	30.3 ± 7.9	30.0 ± 7.8	33.3 ± 7.2
Mean disease duration (years)	—	—	—	1.5 ± 1.0	1.5 ± 1.1	3.6 ± 1.1
Hoehn and Yahr stage	0.1 ± 0.6^b^	0.2 ± 0.6	0.1 ± 0.4	2.0 ± 0.2	2.0 ± 0.2	2.0 ± 0.1
Levodopa‐equivalent daily dose (mg)	—	—	—	309.0 ± 257.9	320.5 ± 290.8	487.4 ± 268.5
Cohort II						
Number of subjects	23	—	—	41	30	30
Mean age (years)	59.7 ± 8.3	—	—	61.1 ± 9.4	60.9 ± 9.8	63.3 ± 9.8
Male/female	12/11	—	—	27/14	20/10	20/10
MDS‐UPDRS‐III OFF score^c^	—	—	—	38.8 ± 10.8	37.2 ± 11.4	—
Mean disease duration (years)	—	—	—	9.3 ± 3.7	9.1 ± 3.9	11.0 ± 4.3
Hoehn and Yahr stage	‐	‐	‐	2.0 ± 0.6	2.0 ± 0.6	2.2 ± 0.3
Levodopa‐equivalent daily dose (mg)	‐	‐	‐	761.3 ± 292.1	738.4 ± 274.3	796.4 ± 301.0

HV, healthy volunteers; PD, patients with Parkinson's disease.

Groups were compared using t‐test and sex was compared using Chi‐2.

^a^ Significant difference between all HV at V1 and all PD at V1 with *P* < 0.05.

^b^ Significant difference between all HV at V1 and all PD at V1 with *P* < 0.001.

^c^ MDS‐UPDRS‐III scores obtained by converting the UPDRS‐III scores.

Data represented as mean **±** standard deviation.

1. Cohort I: Ninety‐nine early PD patients and 41 HVs were analyzed at V1, of whom 79 PD patients and 32 age‐matched HVs were analyzed at both V1 and V2 with an average of 2.0 ± 0.2 years of follow‐up. There was no significant difference in age between HV and PD. There was a larger proportion of men among patients at baseline (χ^2^ = 7.630, *P* = 0.005).
2. Cohort II: Forty‐one progressing PD patients and 23 HVs were analyzed at V1, of whom 30 PD patients had both V1 and V2, with an average of 2.4 ± 0.5 years follow‐up. There were no significant differences in age and sex proportions between groups.

### 2.2 Imaging Results

#### 2.2.1 Cross‐Sectional (Table 2)

**TABLE 2:** SN measurements

(a) Baseline: GLM‐ANOVA with group (PD, HV) as between‐group factors adjusted for age and sex as covariates												
			Healthy volunteers		Parkinson's disease patients				GLM‐ANOVA: group factor			
Baseline			All HV		All PD		% Change		*F*		*P*	
Cohort I			n = 41		n = 99							
Volume (mm^3^)			273.2 ± 48.4		242.5 ± 50.2^b^		−11.2		11.42		**< 0.001**	
Corrected volume (C_vol_)			0.19 ± 0.04		0.16 ± 0.04^b^		−16.1		24.22		**< 0.001**	
Signal‐to‐Noise Ratio (SNR)			112.1 ± 1.6		110.0 ± 1.6^b^		−17.4		47.95		**< 0.001**	
Contrast‐to‐noise ratio (CNR)			1.56 ± 0.23		1.25 ± 0.27^b^		−20.3		48.26		**< 0.001**	
Cohort II			n = 23		n = 41							
Volume (mm^3^)			260.4 ± 37.0		160.3 ± 63.6^b^		−38.4		43.57		**< 0.001**	
Corrected volume (C_vol_)			0.19 ± 0.03		0.11 ± 0.05^b^		−42.5		53.55		**< 0.001**	
Signal‐to‐noise ratio (SNR)			109.8 ± 2.4		107.7 ± 2.1^b^		−21.4		13.46		**< 0.001**	
Contrast‐to‐noise ratio (CNR)			1.30 ± 0.39		1.01 ± 0.29^b^		−22.7		10.76		**< 0.01**	

(b) Longitudinal: GLM‐ANOVA with visit (V1, V2) as a within‐subject factor and group (PD, HV) as between‐group factor adjusted for age and sex as covariates												
							GLM‐ANOVA					
	Healthy volunteers			Parkinson's disease patients			Group factor		Visit factor		Group X visit	
Longitudinal	V1	V2	Annual rate of decline (%)	V1	V2	Annual rate of decline (%)	*F*	*P*	*F*	*P*	*F*	*P*
Cohort I												
Volume (mm^3^)	266.8 ± 51.3	268.9 ± 51.7	0.4	242.8 ± 49.2	214.1 ± 53.6^b^	‐5.8	26.91	**< 0.001**	8.36	**0.004**	4.12	**0.043**
Corrected volume (C_vol_)	0.19 ± 0.04	0.19 ± 0.04	0.3	0.16 ± 0.04	0.14 ± 0.04^b^	‐5.6	54.96	**< 0.001**	7.96	**0.005**	3.67	*0.056*
Signal‐to‐noise ratio (SNR)	112.2 ± 1.8	112.4 ± 1.6	0.8	110.0 ± 1.7	110.3 ± 1.7^a^	1.5	73.53	**< 0.001**	1.60	0.200	0.02	0.880
Contrast‐to‐noise ratio (CNR)	1.58 ± 0.24	1.71 ± 0.25^b^	3.9	1.26 ± 0.29	1.31 ± 0.27^a^	1.9	90.38	**< 0.001**	4.15	**0.042**	1.00	0.322
Cohort II												
Volume (mm^3^)				157.6 ± 69.1	119.0 ± 60.2^b^	−10.2			5.32	**0.024**		
Corrected volume (C_vol_)				0.11 ± 0.05	0.08 ± 0.04^b^	−10.3			5.04	**0.024**		
Signal‐to‐noise ratio (SNR)				107.8 ± 2.1	107.1 ± 1.6^a^	−3.7			1.87	0.200		
Contrast‐to‐noise ratio (CNR)				1.03 ± 0.30	0.96 ± 0.24	−3.0			1.07	0.300		

HV, healthy volunteer; PD, patients with Parkinson's disease.

Data represented as mean ± standard deviation.

Significant *P* values are indicated in bold.

^a^ t‐Test *P* < 0.05.

^b^ *t*‐Test *P* ≤ 0.001.

1. Cohort I: All SN measurements significantly differed between early PD patients and HVs at V1 (Table 2). ROC analysis provided area under the curve (AUC) of 0.670 for Vol, 0.728 for C_vol_, 0.835 for SNR, and 0.830 for CNR. C_vol_ was significantly larger in women than in men in both groups (Table S2).
2. Cohort II: As in early PD, all SN measurements significantly differed between progressing PD patients and HVs at V1 (Table 2). ROC analysis provided AUC of 0.916 for Vol, 0.929 for C_vol_, 0.745 for SNR, and 0.736 for CNR. C_vol_ was also larger in women than in men in both groups. There was a significant interaction between group and sex for both SNR and CNR, higher in women than in men in HVs (Table S2).

#### 2.2.2 Longitudinal (Table 2)

**FIG. 2:**
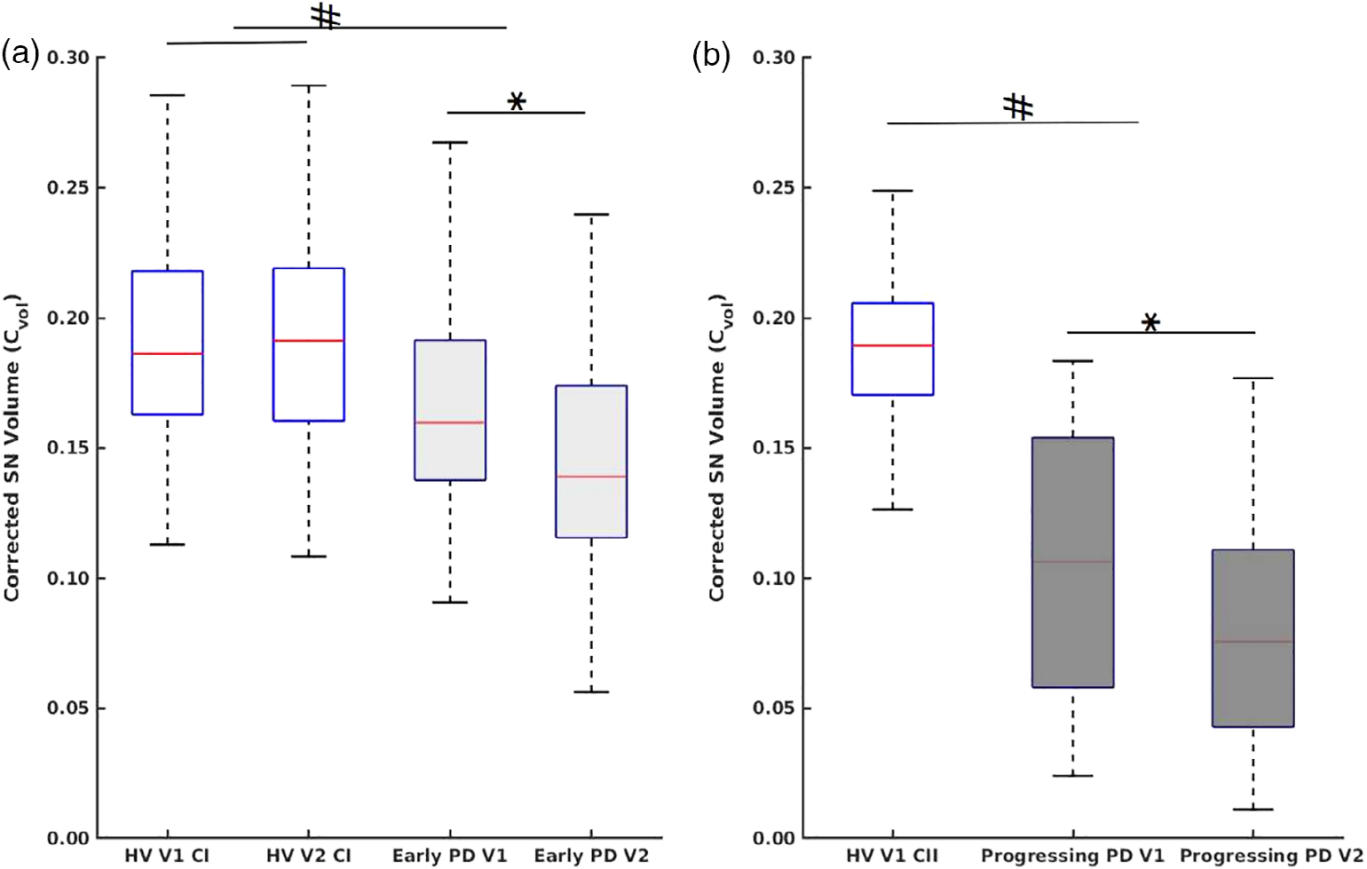
(a) Box plots of C_vol_ at longitudinal (V1) and follow‐up (V2) in HVs (cohort I [CI]) and early PD. (b) Box plots of C_vol_ at longitudinal (V1) and follow‐up (V2) in HVs (cohort II [CII]) and progressing PD patients. Disease severity is indicated using darker shades of gray, ^#^ *P* < 0.001 (ANOVA), *P < 0.001 (*t* test). [Color figure can be viewed at wileyonlinelibrary.com]

1. Cohort I: Vol and C_vol_ showed significant effect for group and visit factors, with a significant reduction in Vol and C_vol_ in early PD compared with HV and between V1 and V2 in PD but not in HV and a significant group × visit interaction for Vol and a trend for C_vol_ (Table 2, Fig. 2). SNR and CNR showed a significant effect for the group factor, with a significant increase in PD compared with nonsignificant increase in HV. There was no significant visit effect for SNR or group × visit interaction for both SNR and CNR (Table 2). There was a significant effect of sex for C_vol_ and CNR, with a decrease in C_vol_ and increase in CNR greater in men than in women in PD and a significant interaction (group × sex) for CNR (Table S2).
2. Cohort II: In progressing PD patients, Vol and C_vol_ demonstrated a significant effect for the visit factor, with a significant reduction between V1 and V2 (Table 2, Fig. 2). There was a nonsignificant decrease in SNR and CNR between V1 and V2 (Table 2). Vol and C_vol_ were significant for sex, with greater values in female PD patients and no visit × sex interaction (Table S2).

#### 2.2.3 Annual rate of changes (Table 2, Table S2)

Cohort I: For early PD, average annual rates of changes were − 5.8% for Vol, −5.6% for C_vol_, 1.5% for SNR, and 1.9% for CNR s compared with a nonsignificant 0.4% for Vol, 0.3% for C_vol_, 0.8% for SNR, and 3.9% for CNR in HV.

Cohort II: For progressing PD, average annual rates of changes were − 10.2% for Vol, −10.3% for C_vol_, and − 3.7% for SNR while nonsignificant, −3.0%, for CNR.

### 2.3 Variability of Measurements

There was a high reproducibility between the measurements performed by the 2 examiners (DICE interobserver, 0.82; DICE intraobserver, 0.85: ICC for Vol, 0.78; ICC for SNR, 0.89).

In HV at baseline, there was a significant scanner effect in SN measurements for Vol (*P* = 0.02), SNR (*P* < 0.001), and CNR (*P* = 0.01), although both groups were matched for age and sex, and no significant difference was found for C_vol_ measurements (*P* = 0.2) between HV groups of cohorts I and II (Table 2). There were significant differences in SN Vol (9.4%), SNR (15.2%), and CNR (13.0%) and nonsignificant difference of in C_vol_ (6.8%) between measurements of cohort I and cohort II. Overall, cohort I demonstrated higher average values than cohort II for all measurements. There was no scanner effect detected on TIV.

### 2.4 Correlations With Age and Clinical Status

Pearson's correlation coefficients between SN measurements, age, and clinical scores showed the following results (Table S1).

1. Correlations with age. Age in early PD correlated negatively with Vol and C_vol_, positive correlation with SNR, and trend for positive correlation with CNR (Table S1). In all other groups, age did not correlate with any SN measurements.
2. Correlations with disease duration and severity. At baseline for early PD, MDS‐UPDRS‐III OFF score had a significantly negative correlation with SN Vol and C_vol_ (Fig. S1, Table S1). Disease duration also correlated negatively with SNR and CNR in early PD, and there was a trend for a negative correlation with SNR in progressing PD.

There were no significant correlations at baseline either for the HV group of cohort I or for the progressing PD and HV groups of cohort II. There were no significant correlations between SN measurements and longitudinal changes in disease severity or time between V1 and V2 for both the cohorts. However, for progressing PD in cohort II, longitudinal changes in disease duration had close to significantly positive correlations with CNR. Although disease duration between visits in early PD or time between visits in HV in cohort I were not correlated.

Correlations with levodopa‐equivalent daily dose (LEDD). At baseline, no correlations were found between LEDD and SN measurements either in early PD (−0.148 < *r* < −0.060, all *P* > 0.05) or in progressing PD (−0.260 < *r* < −0.115, all *P* > 0.05). Similarly, there were no correlations in longitudinal changes in either early PD (−0.057 < *r* < 0.046, all *P* > 0.05) or in progressing PD (−0.030 < *r* < −0.029, all *P* > 0.05); see Table 1 and Table S1.

### 2.5 Sample Size Estimation

To detect an effect size of 30% to 70% over 1‐year follow‐up in early PD, sample size needed for the treatment group was lower for volume measurements (corrected for TIV or not) than for signal intensity measurements based on CNR. For Vol and C_vol_, sample size ranged from 20 (effect size, 70%) to 120 (effect size, 30%) subjects. For SNR and CNR, sample size ranged from 40 (effect size, 70%) to 500 (effect size, 30%) subjects (Table 3, Fig. S2).

**TABLE 3:** Estimation of sample size needed to detect 1‐year changes in neuroimaging end points for clinical trials

	Volume (Vol, mm^3^)	Corrected volume (C_vol_)	Signal‐to‐noise ratio (SNR)	Contrast‐to‐noise ratio (CNR)
30% effect of drug				
Sample size 90% power	118	120	500	440
80% power	90	95	400	320
50% effect of drug				
Sample size 90% power	60	60	160	120
80% power	40	40	120	90
70% effect of drug				
Sample size 90% power	30	30	90	60
80% power	20	20	60	40

## 3 Discussion

This study suggests that NM‐sensitive imaging provides reliable progression markers of SN neurodegeneration. First, we confirmed that NM‐based volume and signal intensity were reduced in PD and that this reduction was greater in progressing than in early PD. Second, we characterized the pattern of progression in NM‐based SN volume and signal intensity in PD patients compared with HVs. The results also suggested that using our segmentation method, volume measurements were more effective than signal changes in detecting longitudinal changes in SN. Third, these changes were clinically relevant as they correlated with the clinical severity of disease assessed using MDS‐UPDRS for SN volumes and disease duration for SN signal changes. Fourth, LEDD in patients did not correlate with any SN measurements, suggesting that NM signal changes were not modified by dopaminergic medication. Last, power analysis suggested that NM‐based SN measurements could be effective in detecting significant changes in a neuroprotective clinical trial. Altogether, the results demonstrated the potential of NM as a useful imaging biomarker of PD progression.

NM is a pigment produced in the cytosol of catecholaminergic neurons stored in NM autophagic lysosome organelles in which it is bound to metals, particularly iron.^9^ The NM–iron complex can be found in an extracellular compartment after neuronal death.^9^ The NM–iron complex is paramagnetic and hence can be detected using MRI and appears bright on turbo or fast spin echo T1‐weighted images. Studies have shown that the area of high signal intensity in these images is associated with the NM containing area of SNc at 3 T^10^, ^15^ and 7 T.^11^, ^14^, ^25^

The relationships between iron and NM in the SN in PD are also of interest. Iron is involved in the metabolic pathway leading to NM synthesis, and the accumulation of iron when not stored in NM may contribute to neurodegeneration in PD.^26^, ^27^ In contrast, the NM–iron complex may have a neuroprotective effect.^9^, ^26^, ^27^ Iron‐sensitive MRI techniques have shown raised iron levels in the SN in PD patients.^28^ A recent study suggested that MRI may help to distinguish NM‐iron from the other iron pool, but this requires further study.^17^, ^29^

SN volume appears highly variable when measured using MRI.^11^, ^12^, ^30^, ^31^ Many factors contribute to this variability such as the type of sequence that was used including T2*‐weighted imaging at 7 T,^32^ driven equilibrium single‐pulse observation of T1,^33^ Fast Gray Matter Acquisition T1 Inversion Recovery,^34^ DANTE T1‐SPACE,^35^ and short‐echo‐time magnitude image derived from quantitative susceptibility mapping,^11^, ^12^, ^23^, ^31^, ^36^, ^37^ Previous studies using NM‐sensitive images have also measured variable SN volumes ranging from about 110 to 500 mm^3^.^11^, ^12^, ^23^, ^30^, ^31^ These measurements depended on the method used for volume calculation, the number of image slices used for SN segmentation, and the imaging parameters such as slice thickness, which determined the amount of partial voluming in images. In studies that used semiautomated thresholding, volumes depended on the choice of threshold, which resulted in smaller (<200 mm^3^),^11^ intermediate (250–350 mm^3^),^12^, ^30^ or larger volumes, that is around 500 mm^3^.^31^ Our values were in the intermediate range, in line with previous studies,^12^, ^23^, ^30^, ^32^, ^38^ Values reported using MRI were similar^39^ or greater than those reported using stereological histological methods.^40^

Baseline reductions in volume and signal intensity were observed in early and progressing PD. In early PD, NM‐based SN volume decreased by 11.2% and SNR by 17.4% compared with HV. Some previous studies reported greater reductions in size, of 22% to 29% in de novo patients^41^, ^42^, ^43^, ^44^ and of 29% to 47% in early PD, either using measurements of SN width or area.^11^, ^30^, ^42^ Volume reductions were more pronounced at later stages of disease, with a 38.4% decrease here compared with 30%–39% reported in moderate PD^12^, ^23^, ^45^ and 78% in late PD.^11^ This decrease was also consistent with the 45% loss reported during the first decade of PD in histology studies.^3^ Signal reductions also greatly varied across studies ranging from −21% in de novo PD^43^ to −48% in late PD.^46^ Studies have also reported greater changes in the lateral part of this structure.^39^, ^43^, ^44^, ^47^, ^48^, ^49^, ^50^, ^51^

Longitudinal reductions in volume and signal intensity were observed in early and progressing PD. Volume decreased by 5.8% per year in early PD patients and by 10.2% per year in late PD patients. One study reported a 17.5% yearly reduction in SN area^18^ and a 16.5% reduction in CNR in PD patients with a 3.1‐year disease duration.^18^ Longitudinal imaging studies of DA function using radiotracers in PD patients have shown an annual decline rate of DAT binding in the striatum of 4.6% to 11.9% compared with baseline values.^52^ Some studies have reported that DAT binding decreased linearly^53^, ^54^, ^55^, ^56^, ^57^ and others exponentially.^56^, ^58^, ^59^, ^60^, ^61^, ^62^, ^63^ Exponential decay of vesicular monoamine transporter was also reported.^64^ Although our results suggest a greater decrease in NM content in progressing stages compared with early stages of disease, the dynamics of longitudinal changes in NM in PD, whether linear or exponential, require further investigation.^2^, ^3^, ^4^, ^5^, ^6^, ^7^, ^8^, ^50^ Overall, the rate of decline in NM‐based SN volume is in line with the values of DAT binding reported in the literature. Although NM is also found in the extracellular space following DA neuronal death before being degraded,^9^ measurement by NM‐sensitive MRI may provide markers more closely related to SN neurodegeneration than striatal DA function^9^ or free water.^65^ This remains to be confirmed by a direct comparison of the different markers and with postmortem studies.

There were significant differences between men and women in both groups. In the HV group, women had greater SN‐normalized volumes (C_vol_) and signal intensities (CNR) and showed greater increase in NM content at V2 compared with men. Larger SN C_vol_ in female HVs was in line with a previous study.^66^ This was also in line with DAT studies in HVs that have reported higher striatal binding in women compared with men.^67^, ^68^, ^69^ The greater increase in NM content with age in women may explain this discrepancy. In PD, women presented larger baseline volumes and lower decrease in volume and increase in signal intensity compared with men. This was also in line with the higher striatal dopamine transporter binding in women compared with men reported in PD.^68^

In early PD at baseline, SN volume changes correlated with disease severity and signal intensity changes with disease duration, in line with correlations reported between motor severity and SN volume or signal intensity^30^, ^39^, ^45^, ^48^, ^70^, ^71^ as well as fractional anisotropy in NM‐based SN^72^ and free water in the posterior SN.^65^ No correlations were observed in progressing PD patients, possibly because of the smaller number of subjects.

There were no correlations between NM SN measures in HVs and age, in line with previous studies that showed that SN NM content reached a plateau in the fifth and sixth decades.^66^ This may also apply to progressing PD patients in the same age range. However, there was a significant effect of age on SNR in early PD, as it is possible that increased production of NM may be associated with greater neuronal loss.^73^

There was a significant scanner effect for SN volume and signal intensity in HVs. This may be because of the differences in sequence parameters, coils, or scanner hardware, resulting in different SNRs. However, head size normalization (C_vol_) cancelled the between‐scanner differences in SN volume and hence should be considered for multisite trials investigating NM signal changes in PD patients. Alternatively, a scanner may be entered in the statistical comparison as a covariate.

Sample size estimates were calculated for detecting a slowing of NM decrease in a clinical trial testing a disease‐modifying therapy. These results are comparable with [^123^I] FP‐CIT SPECT or MRI‐based free water measurements.^65^

This study has several limitations. First, we used manual segmentation to delineate the SN. Automated methods may improve the reproducibility of segmentation techniques.^74^, ^75^, ^76^, ^77^ Manual segmentation has the advantage of allowing careful quality control of images and removal of areas containing artifacts from measurements. Experienced raters can achieve good reproducibility of measurements, as it was in our case, in line with those reported in previous studies.^18^, ^23^, ^30^, ^39^, ^44^, ^45^, ^46^, ^48^, ^66^ Second, 2‐dimensional acquisitions using a relatively thick slice are prone to partial voluming, and the use of 3‐D acquisitions may improve the accuracy of the results.^78^ Third, improved CNR in SN compared with background signal may be obtained by using different acquisition schemes like magnetization transfer^11^, ^37^, ^72^ or double inversion T1‐weighted imaging, although improved CNR offered by these MRI protocols comes at the expense of acquisition times. Fourth, direct quantitative methods, including T1 mapping^79^ or magnetization transfer, remains to be evaluated.^11^, ^72^ Fifth, the sex ratio in HVs was significantly different from that in the PD patients at baseline, thus further validating that the use of a larger number of subjects with balanced sex ratio is warranted to confirm the results. Last, we converted UPDRS‐III scores to MDS‐UPDRS‐III scores, which may be less accurate than the directly obtained scores as in cohort I.

In conclusion, this study showed a progressive and measurable decrease in NM‐based SN volume and signal intensity in PD patients over time that related to the severity of motor symptoms. NM‐sensitive imaging might allow direct, noninvasive assessment of the progression of SN cell loss in PD. In clinical trials, NM‐based SN volume measurements could provide useful biomarkers for clinical trials of disease‐modifying therapies.

## Author Contributions

Design, conceptualization and execution of the study, data analysis, statistical analyses and drafting the first and a significant portion of the manuscript, tables and figures: Rahul Gaurav.

Execution of the statistical analyses, review and critique of the manuscript: Lydia Yahia‐Cherif.

Acquisition of clinical data, review and critique of the manuscript: Graziella Mangone.

Execution of the study, data analysis, review and critique of the manuscript: Romain Valabrègue and Nadya Pyatigorskaya.

Review and critique of the statistical analyses and manuscript: Emma Biondetti, Claire Ewenczyk, R. Matthew Hutchison, and Jess M. Cedarbaum.

Design, conceptualization and execution of the study, review and critique of the statistical analyses and manuscript: Jean‐Christophe Corvol and Marie Vidailhet.

Design, organization, conceptualization, and execution of the study, writing of the first draft, overall review and critique of the manuscript: Stéphane Lehéricy.

## Supporting information

**FIG. S1.** Correlation plots between the MDS‐UPDRS‐III (OFF) scores and SN volume (Vol) and SN volume normalized by total intracranial volume (C_vol_).

**FIG. S2.** Plot showing the estimation of sample size needed to detect 1‐year changes in neuroimaging end points for clinical trials where volume is in blue, corrected volume in red, SNR is in yellow, and CNR in violet.

**TABLE S1.** Correlations of SN measurements with disease duration, severity, and age

**TABLE S2**. Effect of sex on SN measurements and total intracranial volume (TIV); baseline: GLM‐ANOVA with group (PD, HV), sex as between‐group factors adjusted for age as a covariate; longitudinal: GLM‐ANOVA with visit (V1, V2) as within‐subject factor and group (PD, HV), and sex as between‐group factors adjusted for age as a covariate
