# Supplementary figures and images for: Longitudinal Changes in Neuromelanin MRI Signal in Parkinson's Disease: A Progression Marker
Source: Mov Disord. 2021 Mar 10;36(7):1592–602. doi: 10.1002/mds.28531 (PMC8359265; doi:10.1002/mds.28531)

**Supplementary Figure 1: Correlation plots**


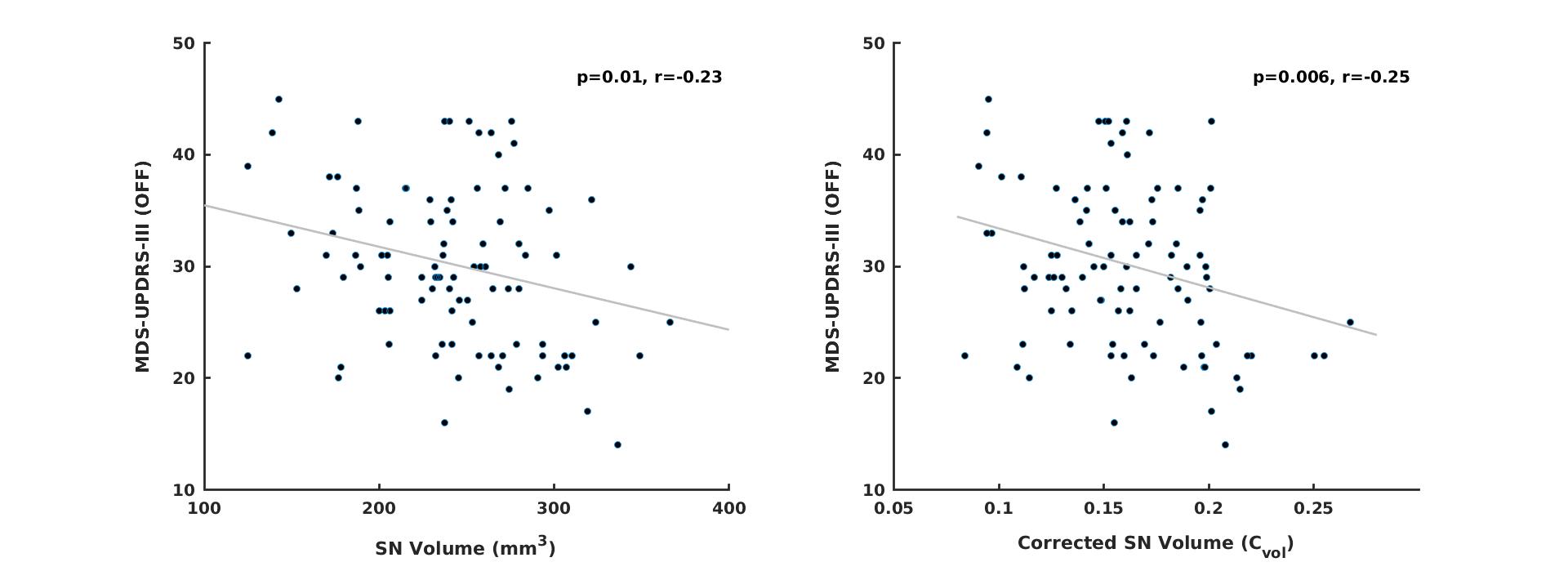

Supplement: Supplementary file 1 — FIG. S1. Correlation plots between the MDS‐UPDRS‐III (OFF) scores and SN volume (Vol) and SN volume normalized by total intracranial volume (Cvol). [file MDS-36-1592-s001.docx]
