# Supplementary material for: Longitudinal Changes in Neuromelanin MRI Signal in Parkinson's Disease: A Progression Marker
Source: Mov Disord. 2021 Mar 10;36(7):1592–602. doi: 10.1002/mds.28531 (PMC8359265; doi:10.1002/mds.28531)
Supplement: Supplementary file 2 — FIG. S2. Plot showing the estimation of sample size needed to detect 1‐year changes in neuroimaging end points for clinical trials where volume is in blue, corrected volume in red, SNR is in yellow, and CNR in violet. [file MDS-36-1592-s004.docx]

**Supplementary Figure 2: Plot showing the estimation of sample size needed to detect 1-year changes in neuroimaging endpoints for clinical trials**


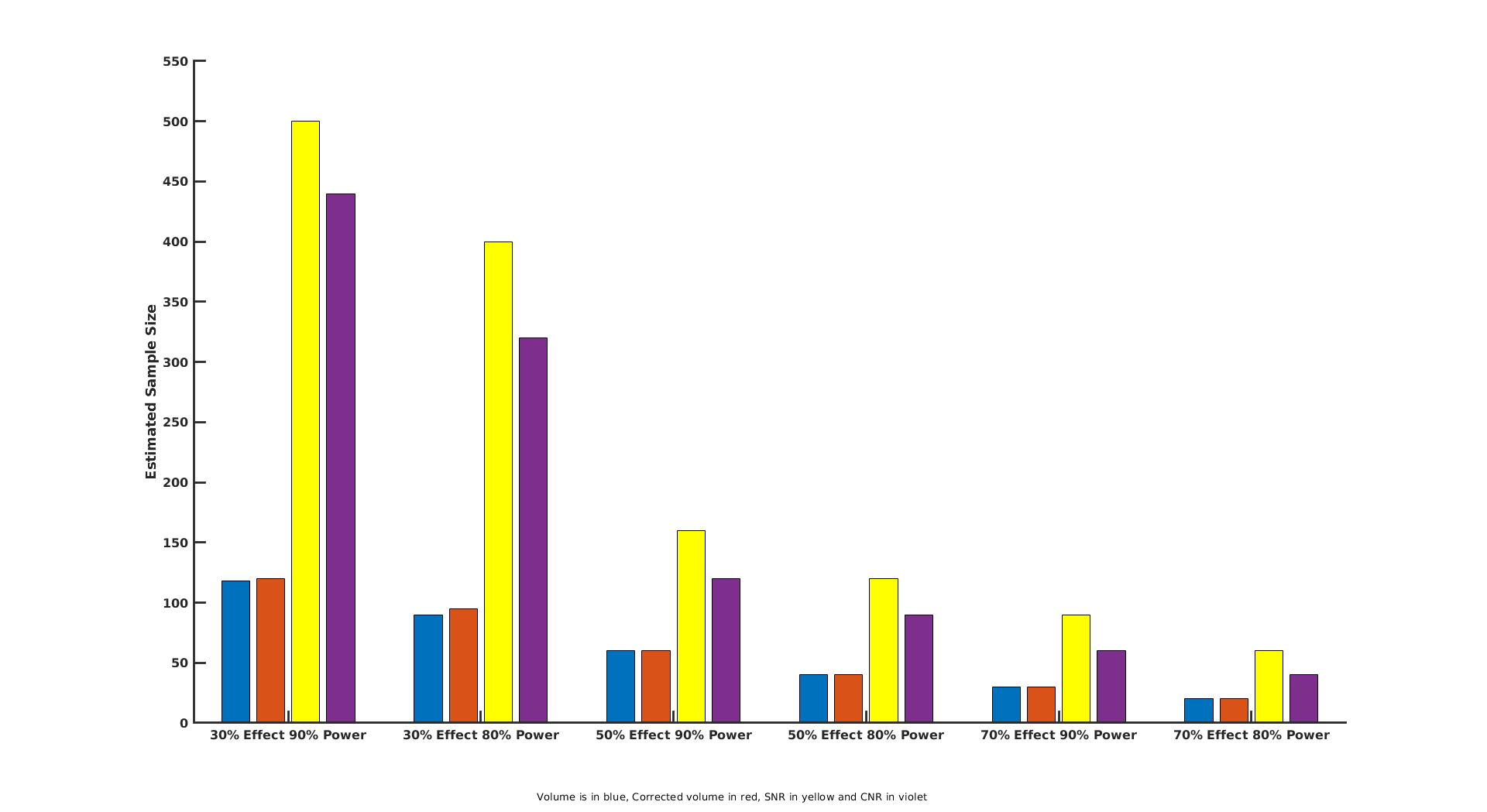
Volume is in blue, Corrected volume in red, SNR in yellow and CNR in violet
