# Supplementary material for: Longitudinal Changes in Neuromelanin MRI Signal in Parkinson's Disease: A Progression Marker
Source: Mov Disord. 2021 Mar 10;36(7):1592–602. doi: 10.1002/mds.28531 (PMC8359265; doi:10.1002/mds.28531)
Supplement: Supplementary file 3 — TABLE S1. Correlations of SN measurements with disease duration, severity, and age [file MDS-36-1592-s003.docx]

**Supplementary Table 1: Correlations of SN measurements with disease duration, severity and age**

|  |  | **Volume (Vol, mm^3^)** | | | **Corrected Volume (C_vol_)** | | | | **Signal-to-Noise Ratio (SNR)** | | | | **Contrast-to-Noise Ratio (CNR)** | | |
| --- | --- | --- | --- | --- | --- | --- | --- | --- | --- | --- | --- | --- | --- | --- | --- |
|  |  | *p* value | | r-value | *p* value | | r-value | | *p* value | | r-value | | *p* value | | r-value |
| **Correlations between clinical and imaging measures at baseline** | | | | | | | | | | | | | | | |
| **Cohort I** | |  | |  |  | |  | |  | |  | |  | |  |
| PD | MDS-UPDRS-III OFF | **0.011** | | -0.238 | **0.006** | | -0.252 | | 0.487 | | -0.002 | | 0.227 | | -0.082 |
|  | Disease duration | 0.396 | | -0.035 | 0.457 | | -0.010 | | **0.003** | | -0.268 | | **0.001** | | -0.281 |
|  | Levodopa equivalent daily dose | 0.097 | | -0.148 | 0.150 | | -0.108 | | 0.210 | | -0.081 | | 0.265 | | -0.060 |
|  | Age | **0.002** | | -0.290 | **0.016** | | -0.230 | | **0.026** | | 0.191 | | *0.065* | | 0.139 |
| HV | MDS-UPDRS-III OFF | 0.092 | | -0.225 | 0.268 | | -0.096 | | 0.497 | | -0.007 | | 0.446 | | -0.020 |
|  | Age | 0.138 | | -0.172 | 0.292 | | -0.093 | | 0.505 | | 0.002 | | 0.324 | | 0.073 |
| **Cohort II** | |  | |  |  | |  | |  | |  | |  | |  |
| PD | MDS-UPDRS-III OFF | 0.374 | | -0.065 | 0.318 | | -0.083 | | 0.318 | | -0.064 | | 0.150 | | -0.163 |
|  | Disease duration | 0.398 | | -0.030 | 0.408 | | -0.020 | | *0.050* | | -0.250 | | 0.114 | | -0.200 |
|  | Levodopa equivalent daily dose | 0.244 | | -0.115 | 0.222 | | -0.126 | | *0.060* | | -0.260 | | 0.111 | | -0.211 |
|  | Age | 0.116 | | -0.170 | 0.282 | | -0.110 | | 0.454 | | 0.020 | | 0.334 | | -0.070 |
| HV | UPDRS-III OFF | 0.456 | | -0.040 | 0.124 | | 0.270 | | 0.472 | | 0.020 | | 0.442 | | 0.010 |
|  | Age | 0.278 | | -0.150 | 0.468 | | 0.020 | | 0.280 | | -0.120 | | 0.120 | | -0.270 |
| **Correlations between percentage changes between V1 and V2 in clinical and imaging measures** | | | | | | | | | | | | | | | |
| **Cohort I** | | | |  |  |  | |  | |  | |  | |  | |
| PD | MDS-UPDRS-III OFF | | 0.412 | -0.030 | 0.318 | -0.047 | | 0.351 | | 0.036 | | 0.200 | | 0.087 | |
|  | Disease duration | | 0.461 | -0.008 | 0.470 | -0.004 | | 0.334 | | -0.044 | | *0.065* | | 0.160 | |
|  | Levodopa equivalent daily dose | | 0.368 | 0.046 | 0.421 | 0.034 | | 0.336 | | -0.057 | | 0.348 | | 0.032 | |
|  | Delay between V1 & V2 | | 0.342 | 0.047 | 0.294 | 0.057 | | 0.261 | | 0.072 | | 0.455 | | 0.002 | |
| HV | MDS-UPDRS-III OFF | | 0.312 | -0.098 | 0.339 | -0.083 | | 0.417 | | 0.044 | | 0.240 | | 0.118 | |
|  | Delay between V1 & V2 | | 0.363 | -0.076 | 0.389 | -0.056 | | 0.350 | | 0.037 | | 0.374 | | -0.071 | |
| **Cohort II** | | |  |  |  |  | |  | |  | |  | |  | |
| PD | Disease duration | | 0.502 | 0.020 | 0.440 | 0.040 | | 0.216 | | 0.140 | | *0.054* | | 0.260 | |
|  | Levodopa equivalent daily dose | | 0.450 | -0.030 | 0.464 | -0.029 | | 0.168 | | -0.193 | | 0.167 | | -0.205 | |
|  | Delay between V1 & V2 | | 0.158 | -0.200 | 0.238 | -0.140 | | 0.378 | | 0.060 | | 0.426 | | 0.030 | |

HV indicates Healthy Volunteers and PD indicates patients with Parkinson’s Disease. Significant correlations are indicated in bold. Trends are indicated in italics.
