# Supplementary material for: Longitudinal Changes in Neuromelanin MRI Signal in Parkinson's Disease: A Progression Marker
Source: Mov Disord. 2021 Mar 10;36(7):1592–602. doi: 10.1002/mds.28531 (PMC8359265; doi:10.1002/mds.28531)
Supplement: Supplementary file 4 — TABLE S2. Effect of sex on SN measurements and total intracranial volume (TIV); baseline: GLM‐ANOVA with group (PD, HV), sex as between‐group factors adjusted for age as a covariate; longitudinal: GLM‐ANOVA with visit (V1, V2) as within‐subject factor and group (PD, HV), and sex as between‐group factors adjusted for age as a covariate [file MDS-36-1592-s002.docx]

**Supplementary Table 2: Effect of sex on SN measurements and Total Intracranial Volume (TIV)**

|  |  | **Healthy Volunteers** | | **Parkinson’s Disease**  **Patients** | | | **GLM-ANOVA** | | | | | | | | |
| --- | --- | --- | --- | --- | --- | --- | --- | --- | --- | --- | --- | --- | --- | --- | --- |
| **Baseline** |  | **All HV** | | **All PD** | | | **Group Factor** | | | **Sex Factor** | | | **Group × Sex** | | |
|  |  | **Female** | **Male** | | **Female** | **Male** | | **F value** | ***p v*alue** | | **F value** | ***p* value** | | **F value** | ***p*** **value** |
| **CI** | Volume (mm^3^) | 268.3±45.5 | 279.8±52.7 | | 253.8±47.8 | 236.7±50.9 | | 11.52 | **<0.001** | | 0.92 | 0.34 | | 2.05 | 0.15 |
|  | Corrected Volume (C_vol_) | 0.20±0.04 | 0.18±0.04 | | 0.19±0.04 | 0.15±0.03 | | 24.52 | **<0.001** | | 28.93 | **<0.001** | | 2.67 | 0.10 |
|  | Signal-to-Noise Ratio (SNR) | 112.0±1.7 | 112.2±1.5 | | 110.3±1.9 | 109.8±1.5 | | 48.11 | **<0.001** | | 0.89 | 0.35 | | 1.47 | 0.22 |
|  | Contrast-to-Noise Ratio (CNR) | 1.57±0.25 | 1.55±0.21 | | 1.38±0.31 | 1.18±0.22 | | 49.15 | **<0.001** | | 10.84 | **0.001** | | 3.51 | *0.06* |
|  | TIV (mL) | 1358.9±94.9 | 1544.0±112.5 | | 1366.5±93.0 | 1608.3±118.5 | | 5.08 | **0.02** | | 131.08 | **<0.001** | | 2.37 | 0.13 |
| **CII** | Volume (mm^3^) | 261.0±33.5 | 260.0±41.6 | | 191.8±44.9 | 143.2±66.4 | | 42.57 | **<0.001** | | 7.43 | **0.04** | | 2.38 | 0.13 |
|  | Corrected Volume (C_vol_) | 0.20±0.03 | 0.18±0.03 | | 0.14±0.03 | 0.09±0.05 | | 52.78 | **<0.001** | | 18.07 | **<0.001** | | 1.34 | 0.25 |
|  | Signal-to-Noise Ratio (SNR) | 110.9±1.9 | 108.8±2.4 | | 107.4±2.2 | 107.9±2.0 | | 13.46 | **<0.001** | | 1.83 | 0.18 | | 5.21 | **0.02** |
|  | Contrast-to-Noise Ratio (CNR) | 1.49±0.35 | 1.13±0.35 | | 1.00±0.31 | 1.01±0.29 | | 10.76 | **<0.001** | | 3.44 | *0.06* | | 5.29 | **0.03** |
|  | TIV (mL) | 1355.2±132.8 | 1467.9±110.7 | | 1412.1±130.7 | 1582.1±140.5 | | 7.75 | **0.007** | | 26.01 | **<0.001** | | 0.96 | 0.33 |

| **Longitudinal** | | **Healthy Volunteers** | | | | | | **Parkinson’s Disease Patients** | | | | | | **GLM-ANOVA** | | | | | | | | | | | | | | | | | | |
| --- | --- | --- | --- | --- | --- | --- | --- | --- | --- | --- | --- | --- | --- | --- | --- | --- | --- | --- | --- | --- | --- | --- | --- | --- | --- | --- | --- | --- | --- | --- | --- | --- |
|  |  | **V1** | | | **V2** | | | **V1** | | | **V2** | | **Group** | | | **Visit** | | **Sex** | | | **Group×Visit** | | | **Group****×Sex** | | | **Visit ×Sex** | | | | **Group×Visit×**  **Sex** | |
|  |  | **Female** | **Male** | **Female** | | **Male** | **Female** | | **Male** | **Female** | | **Male** | **F value** | | ***p***  **value** | **F value** | ***p* value** | | **F value** | ***p* value** | | **F value** | ***p* value** | | **F value** | ***p* value** | | **F value** | ***p* value** | **F value** | | ***p* value** |
| **CI** | Volume (mm^3^) | 265.7±47.8 | 268.7±59.5 | 266.7±50.7 | | 272.8±55.7 | 250.9± 44.0 | | 237.6± 52.1 | 187.5±50.0 | | 204.6±56.1 | 26.99 | | **<0.001** | 8.33 | **0.004** | | 3.05 | *0.08* | | 4.13 | **0.04** | | 3.17 | *0.07* | | 0.26 | 0.61 | 0.20 | | 0.65 |
|  | C_vol_ | 0.2±0.04 | 0.18±0.04 | 0.2±0.04 | | 0.18±0.03 | 0.18±0.03 | | 0.15±0.03 | 0.14±0.04 | | 0.13±0.04 | 54.86 | | **<0.001** | 7.94 | **0.005** | | 46.95 | **<0.001** | | 3.66 | *0.056* | | 2.49 | 0.11 | | 0.05 | 0.81 | 0.08 | | 0.77 |
|  | SNR | 111.9± 1.8 | 112.6±1.7 | 112.5± 1.9 | | 112.3± 1.2 | 110.4±1.9 | | 109.8± 1.5 | 112.0±1.8 | | 110.1±1.5 | 73.63 | | **<0.001** | 1.60 | 0.20 | | 2.32 | 0.12 | | 0.02 | 0.88 | | 2.35 | 0.12 | | 0.16 | 0.68 | 0.77 | | 0.38 |
|  | CNR | 1.56±0.26 | 1.62±0.20 | 1.74± 0.29 | | 1.65± 0.17 | 1.39±0.32 | | 1.18±0.23 | 1.69±0.32 | | 1.24±0.21 | 91.70 | | **<0.001** | 4.20 | **0.04** | | 16.66 | **<0.001** | | 1.01 | 0.31 | | 4.77 | **0.02** | | 0.03 | 0.80 | 1.32 | | 0.25 |
|  | TIV (mL) | 1350.4±95.7 | 1539.3±101.4 | 1354.7± 96.4 | | 1541.3±108.2 | 1374.6±91.7 | | 1615.8±123.7 | 1310.3±99.9 | | 1615.3±123.1 | 44.99 | | **<0.001** | 0.01 | 0.91 | | 223.82 | **<0.001** | | 0.00 | 0.93 | | 1.76 | 0.18 | | 0.00 | 0.92 | 0.00 | | 0.99 |
| **CII** |  |  |  |  | |  |  | |  |  | |  |  | |  |  |  | |  |  | |  |  | |  |  | |  |  |  | |  |
|  | Volume (mm^3^) |  |  |  | |  | 200.1±48.7 | | 135.1±68.7 | 147.4±57.5 | | 104.0±57.5 |  | |  | 4.77 | **0.03** | | 6.50 | **0.01** | |  |  | |  |  | | 0.38 | 0.54 |  | |  |
|  | C_vol_ |  |  |  | |  | 0.14±0.03 | | 0.08 ±0.05 | 0.11 ±0.04 | | 0.07±0.04 |  | |  | 5.25 | **0.03** | | 14.11 | **<0.001** | |  |  | |  |  | | 0.61 | 0.44 |  | |  |
|  | SNR |  |  |  | |  | 107.0±2.2 | | 108.1 ±2.0 | 106.8 ±1.7 | | 107.3 ±1.6 |  | |  | 1.72 | 0.20 | | 2.71 | 0.10 | |  |  | |  |  | | 0.31 | 0.58 |  | |  |
|  | CNR |  |  |  | |  | 0.10±0.35 | | 1.05 ±0.28 | 0.98±0.26 | | 0.94±0.24 |  | |  | 0.90 | 0.34 | | 0.1 | 0.80 | |  |  | |  |  | | 0.40 | 0.50 |  | |  |
|  | TIV (mL) |  |  |  | |  | 1403.4±120.7 | | 1575.2±130.3 | 1399.7±125.0 | | 1571.4±135.4 |  | |  | 0.01 | 0.90 | | 27.48 | **<0.001** | |  |  | |  |  | | 0.00 | 0.99 |  | |  |

CI is Cohort I, CII is Cohort II, HV indicates Healthy Volunteers and PD indicates patients with Parkinson’s Disease. Significant correlations are indicated in bold. Trends are indicated in italics.
